# Inter- and intra-observer variability of software quantified bowel motility measurements of small bowel Crohn’s disease: findings from the MOTILITY trial

**DOI:** 10.1186/s13244-025-01978-8

**Published:** 2025-05-27

**Authors:** Maira Hameed, Andrew A. Plumb, Kashfia Chowdhury, Norin Ahmed, Safi Rahman, Gauraang Bhatnagar, Elen Thomson, Maryam Mohsin, Jude Holmes, Steve Halligan, Stuart A. Taylor, on behalf of the MOTILITY trial investigators, Maira Hameed, Andrew A. Plumb, Kashfia Chowdhury, Norin Ahmed, Safi Rahman, Gauraang Bhatnagar, Elen Thomson, Maryam Mohsin, Steve Halligan, Stuart A. Taylor, Tariq Ahmad, Saiam Ahmed, Fardowsa Ahmed-Timms, Rachel Baldwin-Cleland, Uday Bannur Chikkeragowda, Nina Barratt, Teresita Beeston, Anisha Bhagwanani, Stuart Bloom, Darren Boone, Biljana Brezina, Amanda Cetroni, Junaid Choudhury, Bessie Cipriano, Maria Dilawershah, Heather Fitzke, Tracy Foster, James Franklin, Anmol Gangi-Burton, Nicola Gibbons, Edmund Godfrey, Arun Gupta, Ailsa Hart, Emma Helbren, Anthony Higginson, Judith Holmes, Rachel Hyland, Elizabeth Isaac, Ilan Jacobs, Roman Jastrub, Mayamol Joseph, Jaspreet Kaur, Yakup Kilic, Klaartje Bel Kok, Felix Kpodo, Shankar Kumar, Hannah Lambie, Sarah Langlands, Eric Loveday, Sara McCartney, Alex Menys, Peter Mooney, Gordon Moran, Felicia Onoviran, Miles Parkes, Anisha Patel, Jaymin Patel, Kamal Patel, Kamini Patel, Nishant Patodi, Sue Philpott, Richard Pollok, Robert Przemiosolo, Helen Rafferty, Javen Ramsami, Charlotte Robinson, Suzanne Roffe, Lindsay Rogers, Konstantina Rosiou, Naomi Sakai, Abi Seward, Harbir Sidhu, Belinda Theis, Nora Thoua, Damian Tolan, Simon Travis, Anvi Wadke, Lana Ward, Annamaria Wilce, Steven Williams

**Affiliations:** 1 Department of Radiology, University College London Hospitals, London, NW1 2BU UK; 2 Centre for Medical Imaging, University College London, London, W1W 7TS UK; 3 Comprehensive Clinical Trials Unit, University College London, London, WC1V 6LJ UK; 4 Department of Radiology, Epsom and St Helier University Hospitals NHS Trust, Epsom, KT18 7EG UK; 5 Department of Radiology, Frimley Health NHS Trust, Frimley, GU16 7UJ UK; 6 Department of Radiology, St James’s University Hospital, Leeds, LS9 7TF UK; 7 Royal Devon and Exeter NHS Foundation Trust, Exeter, UK; 8 Institute of Medical Imaging and Visualisation Bournemouth, University Bournemouth, Bournemouth, UK; 9 St Mark’s the National Bowel Hospital, London, UK; 10 Translational Medical Sciences, School of Medicine, Faculty of Medicine and Health Sciences University of Nottingham, Nottingham, UK; 11 Department of Gastroenterology, University College London Hospitals NHS Foundation Trust, London, UK; 12 Addenbrookes Hospital, Cambridge, UK; 13 NIHR Nottingham BRC University of Nottingham and Nottingham University Hospitals, Nottingham, UK; 14 Barts and The London NHS Trust, London, UK; 15 Nottingham University Hospitals NHS Trust, Nottingham, UK; 16 Portsmouth Hospitals NHS Trust, Portsmouth, UK; 17 Radiology Department Hull University Teaching Hospital NHS Foundation Trust, Hull, UK; 18 Royal Berkshire NHS Foundation Trust, Reading, UK; 19 Homerton NHS Foundation Trust, London, UK; 20 North Bristol NHS Trust Frenchay Hospital, Bristol, UK; 21 Motilent, London, UK; 22 Radiology Department Western General Hospital, Edinburgh, UK; 23 St George’s University Hospital NHS Foundation Trust, London, UK; 24 Kennedy Institute of Rheumatology, University of Oxford, Oxford, UK

**Keywords:** Crohn disease, Magnetic resonance imaging, Gastrointestinal motility, Observer variation, Biomarkers

## Abstract

**Objectives:**

Motility magnetic resonance imaging (mMRI) is a potential marker of disease activity of small bowel Crohn’s disease (SBCD), but there is limited data on its reproducibility. We assessed inter- and intra-observer agreement of small bowel motility as part of a prospective multicentre trial investigating whether mMRI can predict longer-term response to biologic therapy in active, non-stricturing SB-CD (MOTILITY Trial).

**Methods:**

297 segmental small bowel motility scores from 104 SBCD patients (mean age 38.9 years, 43 female) recruited to the MOTILITY trial were measured independently by two radiologists experienced in mMRI, using GIQuant software. Twenty-six datasets were re-read by both radiologists to test intra-observer variability after a washout period of at least 6 weeks. Five gastrointestinal radiologists inexperienced in mMRI derived 66 segmental motility scores from the same 30 randomly selected patients. Agreement was quantified using the intra-class correlation coefficient (ICC).

**Results:**

There was moderate agreement for mMRI-derived segmental small bowel motility measurements for both mMRI-experienced and inexperienced radiologists (ICC 0.59 (95% CI: 0.51, 0.66) and 0.70 (95% CI: 0.61, 0.78), respectively). Agreement remained moderate to good, combining the experienced trial MRI reader measurements with those of the five inexperienced radiologists (ICC 0.69 (95% CI: 0.61, 0.78). Intra-observer agreement for the two mMRI experienced radiologists was (0.71 (95% CI: 0.44, 0.86) and 0.70 (95% CI: 0.44, 0.86)).

**Conclusions:**

There is moderate to good interobserver agreement for mMRI measurements of segmental small bowel motility for both experienced and inexperienced radiologists.

**Critical relevance statement:**

Study findings support the continuing clinical translation of motility MRI as a reproducible biomarker of disease activity and treatment response in Crohn’s disease.

**Key Points:**

- Motility MRI is a novel biomarker of small bowel Crohn’s disease activity.
- Currently, limited data on intra- and inter-observer variability exists.
- Motility MRI shows moderate to good inter- and intra-observer agreement.
- Intraclass correlation was 0.59–0.71 for experienced and inexperienced radiologists.
- Motility MRI is reproducible, supporting its utility as a biomarker of disease activity.

**Graphical Abstract:**

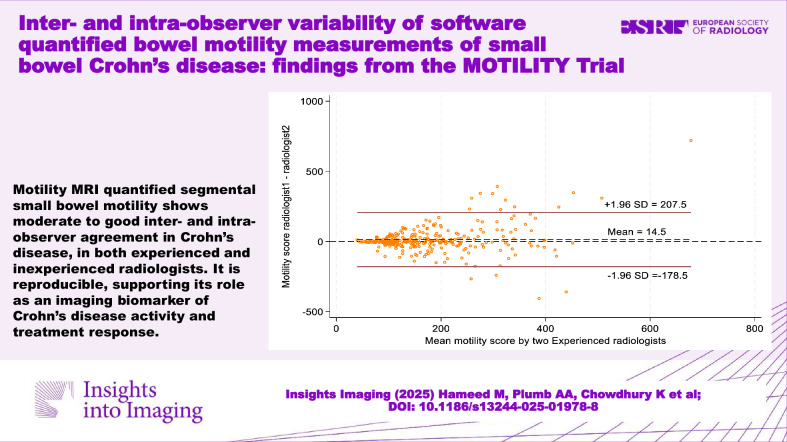

## Introduction

Crohn’s disease (CD) is a chronic, relapsing-remitting disease, most commonly affecting the small bowel. Tight disease control aiming to treat inflammation and heal the bowel, often with biologic therapy, is essential to avoid cumulative, irreversible bowel damage [1, 2]. Evaluating disease activity is fundamental as this guides therapeutic strategy, and response assessment thereafter [3, 4]. Magnetic resonance enterography (MRE) is used widely to diagnose and evaluate CD [3, 5]. Multiple morphological observations have been validated as biomarkers of CD activity, notably bowel wall thickness and T2 signal, mesenteric stranding and signal, and contrast enhancement [2, 3, 5–7]. These observations can be combined into multivariable disease activity scores, such as the London score [8] or Magnetic Resonance Index of Activity (MaRIA) scores [9, 10], which are used mainly for clinical trials. More recently, software quantified segmental bowel motility (motility MRI, mMRI) has been developed as an alternative biomarker of disease activity, demonstrating an inverse correlation with disease activity reported consistently when judged against a range of reference standards, including endoscopic and histopathological [11, 12]. One potential advantage of mMRI is that it may be a more responsive marker of treatment response than morphological observations, which tend to lag behind clinical improvement [13].

Any useful imaging biomarker must demonstrate clinically adequate reproducibility within and between radiologists. While interobserver variation is expected, excessive variation will limit clinical utility in daily practice. Interobserver variation for morphological markers of disease activity is well established. For example, Jairath et al found substantial interrater agreement for the MaRIA, London, and extended London scores when 50 MRE studies were each interpreted three times by four radiologists, although agreement was moderate for individual observations such as wall thickness and T2 signal [14]. To date there has been little research regarding inter- and intra-observability of mMRI, with studies typically using a small number of observers interpreting a small number of datasets [12, 13].

MOTILITY (ISRCTN14481560) was a prospective multicentre trial investigating whether mMRI could predict longer-term response to biologic therapy in active, non-stricturing small bowel CD, compared to C-reactive protein (CRP). The trial provided an opportunity to test inter- and intra-observer variability of mMRI measurements across a range of MRE datasets and radiologists. We investigated the inter-observer variability of mMRI quantified small bowel motility for (i) radiologists experienced in mMRI, and (ii) radiologists experienced in MRE but with limited or no prior mMRI experience. Additionally, we investigated intra-observer variability for the experienced mMRI group.

## Methods

The MOTILITY trial aimed to determine if mMRI was superior to CRP for predicting response and remission at one year in patients commencing biologic therapy for active, non-stricturing small bowel CD; the protocol has been published: https://www.isrctn.com/editorial/retrieveFile/18aadd81-26ad-48d6-ab3e-6d90eb5b2d06/33110. Briefly, patients aged 16 years or older, commencing biologic therapy, were prospectively recruited from 13 UK hospitals. They underwent MRE, including mMRI and CRP at baseline and post-induction (12–30 weeks), with some patients also undergoing a third MRE at around 1 year. The trial was ethically approved (NHS West Midlands Research Ethics Committee: 17/WM/0106) and registered (ISRCTN14481560). The current study is a prespecified sub-study. All study participants included provided informed, written consent as per the study protocol.

### MRE with mMRI protocol and analysis

MRE was performed using standard MRI platforms (1.5 Tesla or greater) and sequences after a 4–6 h fast, and oral contrast was used for the standard MRE. Minimum MRE sequences and acquisition of cine MRI images encompassing the entire small bowel volume are detailed in the supplementary information. mMRI sequences were performed before Buscopan administration.

Images were uploaded onto a cloud-based viewing platform (Entrolytics, Motilent, UK) for subsequent analysis. As part of the main trial protocol, for each MRE dataset, an experienced radiologist (consultant level with experience of > 100 MRE scans and using MRE in day-to-day clinical practice) selected what, in their opinion, was the most active small bowel segment (based on conventional parameters such as wall thickening, mural T2 signal, mesenteric changes etc.) and calculated the London disease activity score: 1.79 + (1.34 × mural thickness score) + (0.94 × mural T2 signal score) [8]. They were blinded to the mMRI sequences.

**Fig. 1:**
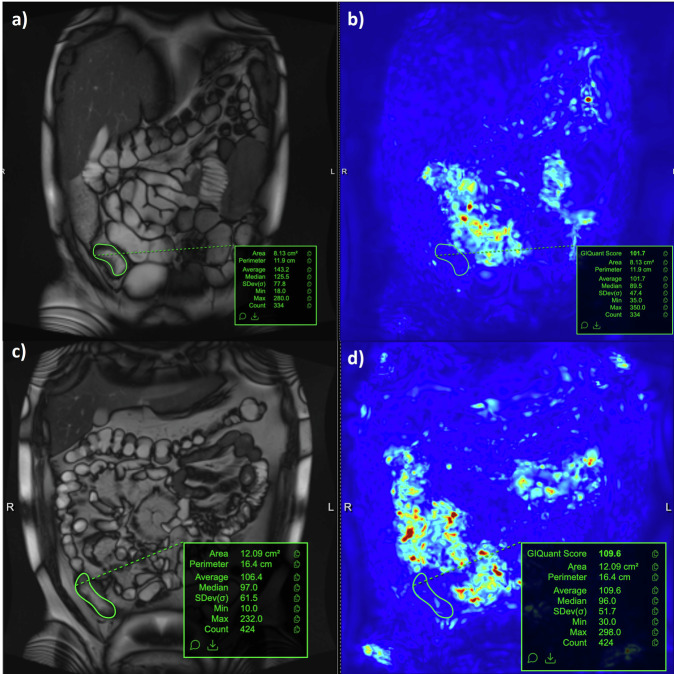
Technique for region of interest (ROI) placement for segmental small bowel motility MRI quantification. **a**, **c** Coronal T2-weighted images showing ROI placement by readers in the terminal ileum in 2 different cases. This ROI placement was selected to be as large as possible but also reproducible between studies at different time points within one patient. These ROIs were transferred to a calculated motility map (**b**, **d**) derived from the reference coronal T2-weighted images. This enables an average motility score to be derived (standard deviation of the Jacobean measured in artificial units)

To quantify motility, MRE scans were processed using a standard software algorithm (GIQuant, Motilent, UK). This produces a reference image for each of the individual 20 s breath-hold motility acquisitions. The user selects the most appropriate reference image for the bowel segment of interest and draws a region of interest (ROI) in the selected bowel segment encompassing as much of the abnormal bowel as possible, including the bowel wall and lumen, but excluding adjacent mesenteric tissues. This ROI is propagated to a motility map to derive a motility score (standard deviation of the Jacobean measured in artificial units, AU, Fig. 1) [15, 16].

For the MOTILITY trial, mMRI measurements were made by one of two radiologists experienced in mMRI (experience of over 500 MREs with motility sequences). For each of the trial patients, one of the radiologists was randomly selected to be the primary study reader, with the second reader independently performing a motility measurement to test interobserver agreement. Both radiologists were informed of which segment (but not the precise location) they should record bowel motility, based on that selected by the central radiologist, calculating the London activity score. The radiologists measuring motility had access to limited anatomical sequences (typically coronal and/or axial T2-weighted or balanced gradient echo sequences) to aid ROI placement. The two experienced radiologists placed ROIs across all the MREs performed for an individual patient at the same sitting, to ensure anatomical registration between ROIs from different patient study visits. A priori, it was agreed that the largest possible ROI should be drawn in the selected small bowel segment that could be best reproduced on both the baseline and post-induction MRE (Fig. 1). Observations were excluded if not all readers could place an ROI on that dataset.

To measure intra-observer variability, after a washout period of at least six weeks, the two mMRI-experienced radiologists repeated the motility measurements in 52 scans (26 each) selected at random by the clinical trial unit (CTU), blinded to their initial ROIs.

To further investigate interobserver variability, five radiologists who were experienced in standard MRE (consultant level, experience of > 100 MRE studies and using MRE in their day-to-day clinical practice) but either no or limited (on average < 20 cases) experience of mMRI measurements, i.e., “mMRI inexperienced” performed motility measurements in 30 patients (each with MRI scans at 3 timepoints) randomly selected by the CTU. The mMRI inexperienced radiologists were provided with an instructional video on how to place the ROI using real case examples recorded by the two mMRI experienced radiologists. The mMRI inexperienced radiologists measured segmental motility using the same protocol as the two mMRI experienced readers from the main trial, as described above.

### Outcome measures

The primary outcome was inter-observer variability for segmental small bowel motility mMRI between (i) radiologists experienced in mMRI and (ii) radiologists experienced in MRE but with limited or no prior mMRI experience. A secondary outcome was the intra-observer variability for mMRI measurements of segmental small bowel motility in the experienced mMRI group.

### Sample size

Previous literature suggests agreement between mMRI experienced radiologists for segmental mMRI measurements is 0.62 [13]. Thirty measurements each made by 5 radiologists and 52 repeated by the same radiologist (intra-observer) permits estimation of intraclass correlation coefficients with 95% confidence interval (CI) width of 0.2 for both inter- and intra-observer agreement [17].

### Statistical analysis

Agreement for the mMRI-derived measurement of small bowel motility in the mMRI experienced and inexperienced groups was quantified using the intra-class correlation coefficient (ICC) and associated 95% CIs. ICC estimates for inter-observer variability were calculated using absolute-agreement, a 2-way random-effects model, and estimates for intra-observer variability were based on absolute-agreement, a 2-way mixed-effects model. Based on the 95% confidence interval of the ICC estimate, values < 0.5, between 0.5 and 0.75, between 0.75 and 0.9, and greater than 0.90 are indicative of poor, moderate, good, and excellent reliability, respectively. [18]. The mean difference and the limits of agreement were also calculated and Bland–Altman plots created for the mMRI experienced radiologists (inter- and intra-observer variation). Scatter plots were also used to observe the mMR experienced radiologist inter-observer and intra-observer agreements.

Statistical analyses were conducted according to a pre-specified statistical analysis plan and performed in Stata/MP 18.0 (StataCorp LLC).

## Results

**Fig. 2:**
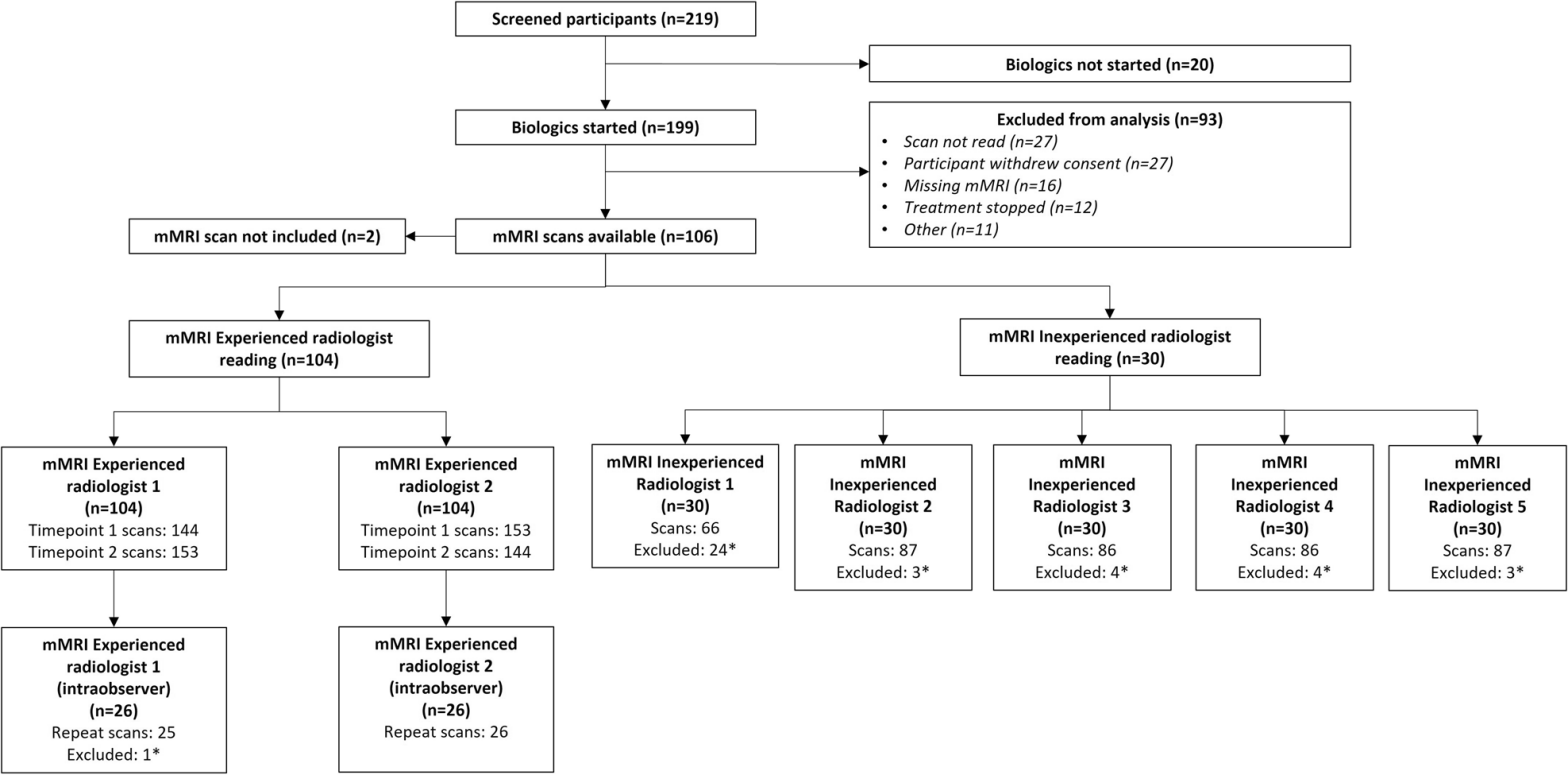
CONSORT diagram of the flow of study participants. mMRI (motility MRI). *Excluded as the reader could not reliably place a region of interest in the study/studies. mMRI inexperienced radiologists were presented 90 scans from 30 patients at 3 time points

Figure 2 illustrates the flow of study participants. Table 1 shows baseline demographics of 104 patients included with active non-stricturing small bowel Crohn’s disease (SBCD), and the 30 patients randomly selected for the mMRI inexperienced group reads. The mean age of the 104 patients was 38.9 years. In 70 (67%), the ileum was the most involved segment, from which mMRI was measured.

**Table 1:** Baseline participant characteristics

Baseline characteristics	Participants assessed by two experienced radiologists *N* = 104		Subgroup of participants assessed by five inexperienced radiologists *N* = 30	
	Mean	(sd)	Mean	(sd)
Age (years)	38.9	13.9	40.1	15.5
MRE score	6.4	1.8	5.8	2.2
	*n*	(%)	*n*	(%)
Gender				
Female	43	41.3	13	43.3
Male	61	58.7	17	56.7
Smoking status				
Non-smoker	49	47.1	15	50
Current smoker	14	13.5	6	20
Ex-smoker	15	14.4	4	13.3
Missing	26	25	5	16.7
Previous bowel surgery				
No surgery	68	65.4	17	56.7
Single surgery	20	19.2	7	23.3
Multiple surgeries	16	15.4	6	20
Presence of stoma				
No	102	98.1	29	96.7
Yes	2	1.9	1	3.3
History of biological therapy				
No	85	81.7	25	83.3
Yes	19	18.3	5	16.7
Age at diagnosis (years)				
A1 (< = 16)	10	9.6	3	10
A2 (17–40)	73	70.2	19	63.3
A3 (> 40)	20	19.2	7	23.3
Missing	1	1	1	3.3
Location				
L1 (ileal)	70	67.3	19	63.3
L3 (ileocolonic)	34	32.7	11	36.7
Behaviour				
B1 (non-stricturing, non-penetration)	55	52.9	15	50
B2 (stricturing)	34	32.7	9	30
B3 (penetrating)	12	11.5	5	16.7
Missing	3	2.9	1	3.3
Perianal disease modifier (*p*)				
No	93	89.4	29	96.7
Yes	8	7.7	0	0
Missing	3	2.9	1	3.3

*n* = the number of participants, *sd* standard deviation, *MRE* magnetic resonance enterography

### Interobserver variability of small bowel motility measurements by mMRI experienced radiologists

**Fig. 3:**
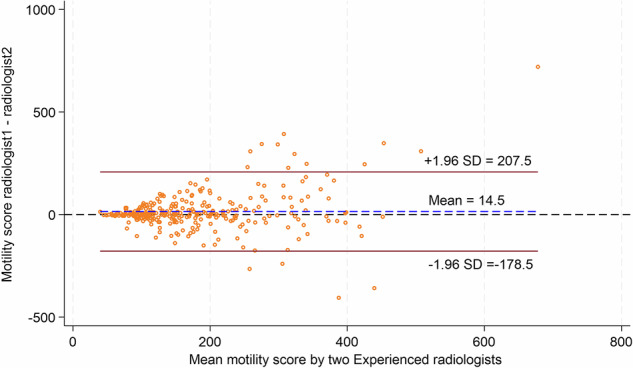
Bland–Altman plot showing interobserver agreement in segmental small bowel mMRI measurements between the two mMRI-experienced radiologists. The red lines represent ± 95% (± 1.96 SD) limits of agreement. SD, standard deviation

**Fig. 4:**
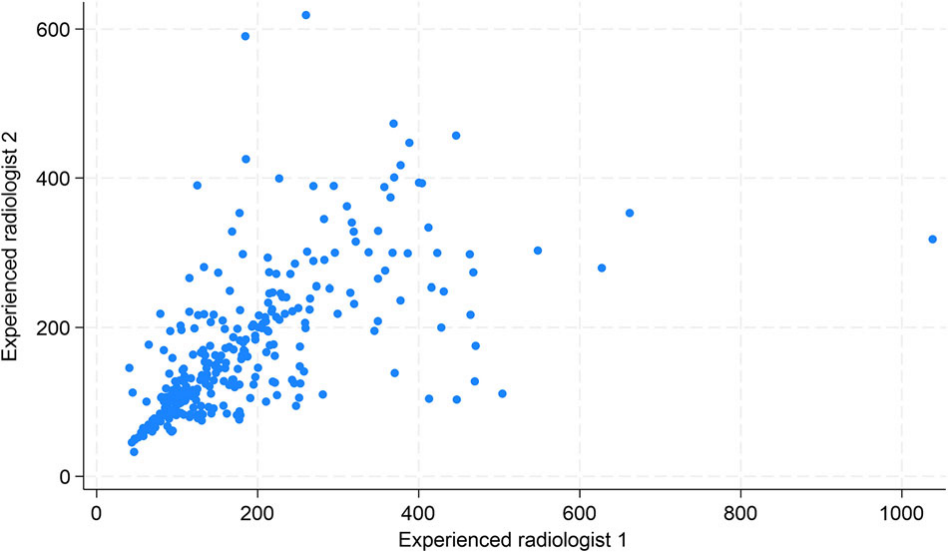
Scatter plot demonstrating mMRI measurement agreement between the two experienced radiologists

297 segmental small bowel mMRI measurements from 104 patients were performed by the two radiologists experienced in mMRI. Table 2 shows that there was moderate inter-observer agreement in this group (ICC 0.59, 95% CI: 0.51, 0.66). A Bland–Altman plot (Fig. 3) shows that the mean difference in mMRI scores between the two experienced radiologists was 14.5 AU with 95% limits of agreement ranging between −178.5 and 207.5. Figure 4 illustrates a scatter plot of agreement of individual mMRI measurements by the 2 readers, showing a tight clustering of values, particularly when scores were below 200 to 250.

**Table 2:** Interobserver variability of segmental small bowel motility measurements for (i) mMRI experienced radiologists (*n* = 2), (ii) mMRI inexperienced radiologists (*n* = 5), and (iii) mMRI inexperienced radiologists with the addition of the primary experienced radiologist readers (*n* = 6)

	Total scans	Mean (SD)	Range	ICC (95% CI)
**Interobserver variability—mMRI Experienced radiologists**				
mMRI Experienced radiologist 1	297	186.6 (120.6)	40.6–1038.2	0.59 (0.51, 0.66)
mMRI Experienced radiologist 2	297	172.1 (97.3)	32.8–618.8	
**Interobserver variability—mMRI Inexperienced radiologists**				
mMRI Inexperienced radiologist 1	66	152.1 (81.3)	37.2–443.2	0.70 (0.61, 0.78)
mMRI Inexperienced radiologist 2	66	152.9 (81.9)	36.4–394.6	
mMRI Inexperienced radiologist 3	66	164.0 (85.0)	36.3–390.9	
mMRI Inexperienced radiologist 4	66	154.6 (81.9)	26.7–389.0	
mMRI Inexperienced radiologist 5	66	143.7 (69.9)	37.0–363.0	
**Interobserver variability—mMRI Inexperienced radiologists plus experienced primary reader**^a^				
mMRI Experienced radiologist	66	161.1 (91.4)	33.3–399.4	0.69 (0.61, 0.78)
mMRI Inexperienced radiologist 1	66	152.1 (81.3)	37.2–443.2	
mMRI Inexperienced radiologist 2	66	152.9 (81.9)	36.4–394.6	
mMRI Inexperienced radiologist 3	66	164.0 (85.0)	36.3–390.9	
mMRI Inexperienced radiologist 4	66	154.6 (81.9)	26.7–389.0	
mMRI Inexperienced radiologist 5	66	143.7 (69.9)	37.0–363.0	

*ICC* intraclass correlation coefficient, *SD* standard deviation, *mMRI* motility MRI

^a^ Addition of the one of two mMRI experienced radiologists used to assess mMRI experienced radiologist inter- and intra-observer variability

### Interobserver variability of small bowel motility measurements by mMRI inexperienced radiologists

A total of 90 scans from 30 patients were allocated to the five mMRI inexperienced radiologists. Twenty-four reads from each radiologist were excluded due to at least one reader’s judgement that the mMRI measurement could not be reliably taken, leaving 66 scans from 24 patients. Table 2 shows that there was moderate to good agreement across 66 mMRI measurements from 24 patients assessed by the five radiologists; ICC of 0.70 (95% CI: 0.61, 0.78). This finding was maintained with the addition of one of the two mMRI experienced trial primary reader measurements; ICC 0.69 (95% CI: 0.61, 0.78).

### Intra-observer variability for mMRI experienced radiologists

**Fig. 5:**
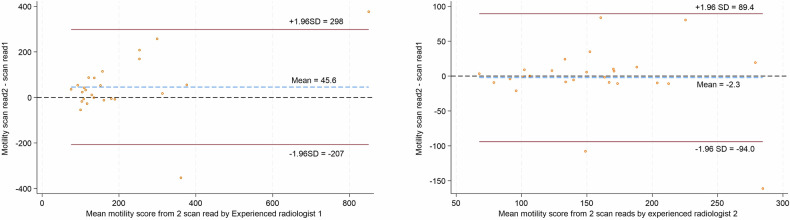
Bland–Altman plot of intra-observer agreement at two time points for mMRI measurements in the experienced radiologists, *n* = 25 for radiologist 1, *n* = 26 for radiologist 2. The red lines represent ± 95% (± 1.96 SD) limits of agreement. SD, standard deviation

**Fig. 6:**
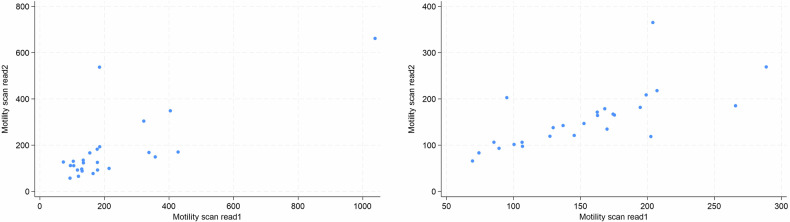
Scatter plots of segmental mMRI measurement intra-observer agreement in the two experienced radiologists between repeat scan reads (reads 1 and 2)

Of the combined 52 scans presented (26 for each radiologist), one was excluded due to a reader’s judgement that the ROI could not be reliably placed based on the available images. There was moderate intra-observer agreement of segmental small bowel mMRI measurements. The ICCs were 0.71 (95% CI: 0.44, 0.86) and 0.70 (95% CI: 0.43, 0.86) across 25 and 26 scans, respectively (Table 3). Bland–Altman and scatter plots also demonstrate good levels of intra-observer agreement in each of the two experienced readers (Figs. 5 and 6). Between the two timepoints, the mean difference in mMRI scores and 95% limits of agreement range was 45.6 AU and −207 to 298, respectively, for reader 1, and −2.3 AU and −94.0 to 84.9 for reader 2. Of note, the maximum value and range of mMRI values were greater for reader 1: 1038.2 and 661.6 AU at timepoints one and two, versus 288.6 and 365.2 AU for reader 2, reflecting the differing datasets allocated to the two readers. There was a tight clustering of values below an average motility score of 200 for both readers.

**Table 3:** Intra-observer variability of segmental small bowel motility measurements for the two mMRI experienced readers at two time points

	Total scans	Mean (SD)	Range	ICC (95% CI)
**Intra-observer variability—mMRI Experienced radiologist 1**				
Primary scan read	25	222.3 (197.6)	72.7–1038.2	0.71 (0.44, 0.86)
Repeat scan read	25	176.8 (144.8)	57.6–661.6	
**Intra-observer variability—mMRI Experienced radiologist 2**				
Primary scan read	26	153.6 (56.0)	69.4–288.6	0.70 (0.44, 0.86)
Repeat scan read	26	155.9 (63.7)	65.9–365.2	

*ICC* intraclass correlation coefficient, *SD* standard deviation, *mMRI* motility MRI

One study was excluded by an experienced radiologist 2

## Discussion

In this prospective, multicentre study of 104 SBCD patients, we found moderate to good interobserver agreement for mMRI-derived measures of segmental small bowel motility, both for radiologists inexperienced and experienced in mMRI. Furthermore, intra-observer agreement was also moderate for two mMRI-experienced radiologists. Bland–Altman analysis and data scatter plots also generally support translation of mMRI to clinical practice with clinically acceptable reproducibility and potential to assess disease activity, for example, when assessing therapeutic response.

Currently, radiologists rely on anatomical MRE observations such as mural thickness and mural and perimural oedema, when making therapeutic response assessments [2, 3, 5–7]. However, recent attention has focussed on functional MRE variables, particularly bowel motility and its incremental value for disease assessment. Quantified terminal ileal motility is more sensitive for disease activity than the MaRIA score when judged against endoscopic and histopathological CD reference standards [12, 13, 15, 19]. Furthermore, informing the design of the MOTILITY trial, initial data suggested mMRI may be better able to capture early response to biologic therapy than morphological observations [13, 20]. It is vital for clinical utility that any promising novel imaging biomarker shows adequate reproducibility within and between readers.

When morphological observations such as bowel wall thickness and T2 signal are combined into disease activity scores, there is relatively good inter- and intra-reader agreement; in 50 MRE studies analysed three times each by four experienced radiologists, Jairath et al found an inter-rater ICC of 0.67–0.71 and intra-rater ICC of 0.87–0.89 for the MaRIA, extended London and London activity scores [14]. However, such activity scores are not used routinely in clinical practice, where radiologists prefer subjective assessment. Notably, the study by Jairath et al, scores for individual anatomical metrics (e.g., mural thickness and mural T2 signal) showed lower agreement.

Conversely, there is relatively sparse published data regarding mMRI inter- and intra-observer agreement, predominantly using a few readers and MRI datasets. Plumb et al found good agreement between two readers at both baseline and follow-up mMRI for SBCD (ICC = 0.65, *p* < 0.001 and ICC = 0.71, *p* < 0.001, respectively) in a single centre, predominantly retrospective study of 46 patients [13]. Dillman et al investigated mMRI in a paediatric and young adult cohort of 20 newly diagnosed SBCD patients starting anti-TNFα therapy and 16 healthy control participants, interpreted by an experienced radiologist but without prior mMRI experience, and a non-medical operator [19]. Terminal ileal motility improved in response to therapy at 6 weeks and 6 months, reported an ICC of 0.89 (95% CI: 0.83–0.93). A study of bowel motility of 15 healthy volunteers found segmental mMRI measurements by one experienced and one inexperienced reader had an ICC of 0.979, *p* < 0.0001 and Bland–Altman limits of agreement 95% CI: −28.9 to 45.9 AU), with an ICC 0.992 and 0.960, *p* < 0.0001) for intra-observer agreement [21].

In the present study, we also found moderate levels of agreement with an intraclass correlation coefficient of 0.59 to 0.70. Specifically, we found that both experienced and inexperienced radiologists exhibited moderate interobserver agreement for segmental small bowel motility, which was maintained when combining the experienced readers’ scores with those of the inexperienced radiologists. Intra-observer agreement for the two mMRI-experienced radiologists was also moderate, although there were wide 95% CI due to a relatively small number of datasets used for this part of the analysis.

Whilst interobserver agreement was apparently higher between readers without experience of mMRI than between those with, the number of measurements made by the experienced readers was almost double that of the inexperienced readers, liking increasing precision around the estimate. Furthermore, the mean MRI motility score from the 30 randomly selected patients testing agreement between inexperienced readers was relatively low, suggesting these datasets included more active (and therefore immotile) disease. ROI placement is easier and less subjective when the bowel is immotile, compared to less inflamed (and more mobile) segments. Indeed, while ICC is commonly used to assess reader agreement, Bland–Altman and raw scatter plots are often more informative as to whether agreement is clinically acceptable, which is dependent on the intended use for the tool. In the present study, the Bland–Altman analysis and scatter plots suggest agreement is lower when bowel with is more motile, usually reflecting normal (responding) bowel; typical mean value of > 220 AU [21]. Further evidence for this observation is the pattern of intra-observer agreement between mMRI experienced radiologists; the datasets of one reader had a low mean motility (and more active disease) with tighter intra-observer agreement than the other. Overall, it is reassuring that agreement was clinically acceptable in the typical range of active disease (< 220 AU), and given that treatment response is predicated by improved motility scores, increased disagreement for bowel approaching normality has less clinical impact.

Our study has several strengths. We included radiologists experienced in MRE interpretation, but not necessarily mMRI, as these are more representative of clinical practice. Our sample size was informed by a power calculation, and a priori, we defined a protocol for ROI placement. Furthermore, to mirror clinical practice, radiologists were provided with limited anatomical sequences to help guide ROI placement and instructed on which small bowel segment to place the ROI. The prospective nature of our study also meant that MRI acquisition protocols could be standardised. While we present ICC data, we also performed Bland–Altman analysis and provide raw scatter plots to better communicate the clinical acceptability around the levels of agreement. Such provisions suggest our results will generalise to standard clinical practice.

There are also some limitations. It is possible that case mix (e.g., disease location and phenotype), influenced mMRI measurements. However, this prospective study included multiple patients from 13 centres (and readers from 3 different centres) and therefore is likely representative of typical clinical practice. While there are many other variables that can be captured with mMRI, such as bowel contractile magnitude and frequency, we focused on one motility metric based on the standard deviation of the Jacobean, as it has a strong evidence base, is simple to perform, and for clinicians and patients to interpret. An ongoing multicentre study is directly assessing the real-world management impact of this single mMRI-derived metric in SBCD on clinical decision making, for both radiologists and gastroenterologists (CONTEXT trial, REC: 21/PR/0592).

In summary, we found that there was moderate to good interobserver agreement for mMRI-quantified segmental small bowel motility in both mMRI-experienced and inexperienced readers. We also found moderate intra-observer agreement in mMRI-experienced readers. This level of mMRI reproducibility is comparable to that of standard MRE morphological variables used in clinical practice. Agreement was best when the bowel was less mobile, i.e., abnormal, which, given the intended use of mMRI, overall supports the ongoing clinical translation of mMRI as a biomarker of disease activity and treatment response in CD.

## Supplementary information

ELECTRONIC SUPPLEMENTARY MATERIAL

## Data Availability

Data reported in the submitted article are held by the central study team based at University College London, following approved data handling and retention policies.

## Abbreviations

AU: Artificial units
CD: Crohn’s disease
CI: Confidence interval
CRP: C reactive protein
CTU: Clinical trial unit
ICC: Intra-class correlation coefficient
MaRIA: Magnetic Resonance Index of Activity
mMRI: Motility MRI
MRE: Magnetic resonance enterography
ROI: Region of interest
SBCD: Small bowel Crohn’s disease
