# Supplementary material for: Inter- and intra-observer variability of software quantified bowel motility measurements of small bowel Crohn’s disease: findings from the MOTILITY trial
Source: Insights Imaging. 2025 May 27;16:111. doi: 10.1186/s13244-025-01978-8 (PMC12116975; doi:10.1186/s13244-025-01978-8)
Supplement: Supplementary file 1 — ELECTRONIC SUPPLEMENTARY MATERIAL [file 13244_2025_1978_MOESM1_ESM.pdf]

Inter- and intra-observer variability of software quantified bowel motility measurements of small bowel Crohn's disease: Findings from the MOTILITY Trial.

ELECTRONIC SUPPLEMENTARY MATERIAL

Insights Imaging (2025) Hameed M, Plumb AA, Chowdhury K, et al.

Supplementary Table 1: MRE minimum anatomical sequences at each time point.

Sequence	T2- Coronal	T2- Axial	T2- Coronal OR Axial
Fat Suppression	No	No	Yes
Example	HASTE	HASTE	FS HASTE

Supplementary Table 2: Target imaging parameters for static and dynamic MRE acquisition, allowing for variation in scanner model. Dynamic MRE sequences were performed before administering Buscopan to avoid impact on quantified segmental small bowel motility scores.

	**Siemens (1.5T)**	**Siemens (3T)**	**Philips (1.5T)**	**Philips (3T)**	**GE (1.5T)**	**GE (3T)**
Vendor name	TrueFISP	TrueFISP	Balanced-TFE	Balanced-TFE	FIESTA	FIESTA
Orientation	Coronal	Coronal	Coronal	Coronal	Coronal	Coronal
Breath Hold	Yes (inspiration)	Yes (inspiration)	Yes (inspiration)	Yes (inspiration)	Yes (inspiration)	Yes (inspiration)
FOV	Variable to cover small bowel	Variable to cover small bowel	Variable to cover small bowel	Variable to cover small bowel	Variable to cover small bowel	Variable to cover small bowel
Slices	Variable to cover small bowel	Variable to cover small bowel	Variable to cover small bowel	Variable to cover small bowel	Variable to cover small bowel	Variable to cover small bowel
Slice thickness	10mm	10mm	10mm	10mm	10mm	10mm
Slice gap	10mm	10mm	10mm	10mm	10mm	10mm
Voxel size	1.5x1.5	1.5x1.5	1.5x1.5	1.5x1.5	1.5x1.5	1.5x1.5
Acquisition Matrix	256x200	256x200	220x221	268x222	256x256	256x244
Flip angle	64	64	70	50	35	50
Fat saturation	None	None	None	None	None	None
TR	3.93	3.93	3	3	3.1	4.2
TE	1.97	1.97	1.5	1.5	1.4	1.8
Temporal resolution	1 second	<1 second	1 second	<1 second	<1 second	<1 second
Dynamic scan time	20 seconds	20 seconds	20 seconds	20 seconds	20 seconds	20 seconds
Number of dynamic stacks	Variable to cover small bowel	Variable to cover small bowel	Variable to cover small bowel	Variable to cover small bowel	Variable to cover small bowel	Variable to cover small bowel
